# Evaluation of Endoplasmic Reticulum Stress in an Experimental Intestinal Ischemia–Reperfusion Model in Rats: The Role of Ozone Therapy and Trimetazidine

**DOI:** 10.3390/biom14091051

**Published:** 2024-08-25

**Authors:** Gokhan Demiral, Tolga Mercantepe, Gurkan Altuntas, Ahmet Pergel, Suleyman Kalcan, Ali Ozdemir, Levent Tumkaya, Sibel Mataraci Karakas, Aykut Ozturk, Adnan Yilmaz

**Affiliations:** 1 Department of General Surgery, Faculty of Medicine, Recep Tayyip Erdogan University, Rize 53100, Turkey; ahmet.pergel@erdogan.edu.tr (A.P.); suleyman.kalcan@erdogan.edu.tr (S.K.); ali.ozdemir@erdogan.edu.tr (A.O.); 2 Department of Histology and Embryology, Faculty of Medicine, Recep Tayyip Erdogan University, Rize 53100, Turkey; tolga.mercantepe@erdogan.edu.tr; 3 Department of Emergency Medicine, Faculty of Medicine, Recep Tayyip Erdogan University, Rize 53100, Turkey; gurkan.altuntas@erdogan.edu.tr; 4 Department of Histology and Embryology, Faculty of Medicine, Ondokuz Mayıs University, Samsun 55010, Turkey; levent.tumkaya@erdogan.edu.tr; 5 Department of Biochemistry, Faculty of Medicine, Recep Tayyip Erdogan University, Rize 53100, Turkey; sibel.mataraci@erdogan.edu.tr (S.M.K.); adnan.yilmaz@erdogan.edu.tr (A.Y.); 6 Department of Pharmacology, Derince Training and Research Hospital, Kocaeli 41900, Turkey; aykut.ozturk@saglik.gov.tr

**Keywords:** endoplasmic reticulum stress, CHOP, GRP-78, intestinal ischemia/reperfusion, oxidative stress, ozone, superior mesenteric artery occlusion

## Abstract

Aim: The objective of the study was to assess the impact of ozone (O_3_) and trimetazidine on the intestines following ischemia–reperfusion (I/R) injury through the investigation of endoplasmic reticulum stress. Methods: Forty Sprague Dawley rats were separated into five groups. The groups were named as follows: control, O_3_, I/R, I/R + trimetazidine (TMZ), and I/R + O_3_. The control group had laparotomy and exploration of the superior mesenteric artery (SMA) only. Furthermore, alongside laparotomy and SMA exploration, an intraperitoneal (i.p.) administration of a 0.7 mg/kg ozone–oxygen (O_3_-O_2_) combination was given to the O_3_ group. In the experimental groups, the SMA was blocked with the silk suture ligation technique for a duration of 1 h and then restored to normal blood flow for another hour. In the I/R + O_3_ group, ozone was delivered i.p. at a dosage of 0.7 mg/kg, 30 min after ischemia. In the I/R + TMZ group, a dose of 20 mg/kg/day of trimetazidine was administered orally via gavage for a duration of 7 days, beginning 1 week prior to the induction of ischemia. Intestinal tissues were taken to assess indicators of intestinal mucosal injury and oxidative stress. Results: The level of the lipid peroxidation marker malondialdehyde (MDA) was significantly reduced in the experimental groups as compared to the I/R group (*p* < 0.05). The experimental groups had considerably greater levels of glutathione (GSH), which reflects antioxidant capacity, compared to the I/R group (*p* < 0.05). Nevertheless, the concentration of GSH was observed to be increased in the I/R + O_3_ group in comparison to the I/R + TMZ group (*p* < 0.05). The histopathological damage score showed a substantial decrease in the experimental groups as compared to the I/R group (*p* < 0.05). The I/R + O_3_ group had the lowest injury score. The experimental groups exhibited significantly reduced positivity of the endoplasmic reticulum (ER) stress markers C/EBP homologous protein (CHOP) and glucose-regulated protein (GRP)-78 compared to the I/R group (*p* < 0.05). Conclusions: The findings provide evidence for the potential advantages of utilizing ozone therapy in the treatment of intestinal ischemia–reperfusion injury. Additionally, they propose that ozone should be assessed in more extensive clinical trials in the future as a therapeutic agent that can disrupt endoplasmic reticulum stress.

## 1. Introduction

Intestinal ischemia–reperfusion (I/R) injury is a significant complication that occurs during surgical procedures and acute mesenteric ischemia. It is a condition where blood flow to the gastrointestinal tract is suddenly interrupted and then restored, resulting in tissue damage [1,2,3,4]. Intestinal ischemia causes damage to the intestinal mucosa, namely due to insufficient oxygen and nutrition supply. This results in cell death and tissue destruction [5]. Reperfusion refers to the restoration of blood flow. Paradoxically, excessive generation of oxygen-dependent free radicals in the ischemic tissue may cause additional damage after the restoration of blood flow [6]. High oxidative stress, tissue necrosis, and systemic inflammation can cause severe issues like multiorgan failure in addition to gastrointestinal system damage [7].

Due to their high metabolic activity, the intestines are especially susceptible to damage during episodes of ischemia and reperfusion [1]. During this phase, an excessive generation of reactive oxygen species (ROS) and proinflammatory cytokines is detected while oxygen is being restored [8]. These compounds have the ability to induce cell damage and apoptosis, resulting in the collapse of the intestinal barrier and ultimately leading to bacterial translocation [5]. Hence, a study in this area holds significant clinical and therapeutic significance.

Extensive research has been carried out in recent years to investigate the molecular processes of intestinal I/R injury [1,9,10,11]. These investigations have resulted in the creation of novel therapeutic approaches aimed at preventing or minimizing harm. Specifically, the application of antioxidant therapy, cytokine inhibition, cooling measures, and pharmaceutical agents has shown encouraging outcomes in animal models [1,5,9,12,13,14].

The endoplasmic reticulum (ER) is crucial for cellular function and survival as it serves as the primary organelle responsible for intracellular protein folding, modification, and transport [15]. Endoplasmic reticulum stress is a response mechanism that occurs when the ER is unable to keep up with the demand for protein folding [16]. Nevertheless, if ER stress becomes severe or prolonged, it can trigger cell death pathways, resulting in major clinical effects in the context of post-ischemia reperfusion injury [17]. ER stress, specifically intracellular calcium imbalances, is strongly linked to heightened oxidative stress and the initiation of the inflammatory response during the ischemia–reperfusion process [18]. Interventions aimed at addressing ER stress may have the potential to provide benefits in minimizing I/R injury [19,20]. Hence, comprehending the impact of ER stress on I/R injury and devising novel treatment approaches to specifically address this mechanism has emerged as a crucial field of study.

Trimetazidine (TMZ) is a medicine that is believed to shield cells from oxidative stress and optimize energy production by regulating mitochondrial energy metabolism [6,21]. Trimetazidine is commonly prescribed to treat heart failure following a heart attack and to avoid angina pectoris in people with coronary artery disease [21]. However, there is new evidence suggesting that TMZ may also have an impact on I/R injury in several organs [22,23].

Ozone (O_3_), a colorless gas, is produced when three oxygen atoms combine and is naturally occurring. It possesses potent oxidative and antibacterial characteristics [24]. Despite its strong oxidative properties, O_3_ has been found to enhance cellular antioxidant responses and decrease oxidative stress when administered in small amounts [13]. The phenomenon that appears to be contradictory is referred to as the “ozone paradox” and provides insight into the positive role of ozone in biological systems. O_3_ possesses qualities that make it appealing due to its capability to eliminate pathogens, enhance blood circulation, improve the ability to carry oxygen, and decrease inflammation by triggering antioxidant defense mechanisms [25]. The beneficial impacts of O_3_ therapy have facilitated its utilization in many medical contexts, particularly in fields such as pain control, circulatory ailments, and immune system regulation [10,25,26,27,28].

Ozone has a lengthy history of therapeutic use in the medical sector and is currently being assessed as a potential treatment for many health disorders [29]. O_3_ has been found to possess anti-inflammatory, antioxidant, and immunomodulatory characteristics, making it a promising candidate for treating cardiovascular and metabolic illnesses [24,27,30]. An essential component of ozone’s medical application is its ability to facilitate tissue repair and regeneration [31,32]. Ozone’s unique characteristics make it a promising candidate for assisting in the treatment of several illnesses, including ischemia–reperfusion injury, wound healing, persistent infections, rheumatoid arthritis, and inflammatory bowel disease [32,33,34,35,36,37,38]. Furthermore, ozone’s antimicrobial properties may contribute to the management of bacterial, viral, and fungal infections, as well as the enhancement of the immune system [28,35,39,40,41,42]. Specifically, research on the impact of O_3_ therapy in reducing I/R injury has shown the therapeutic benefits of this method [13,14,24,43]. O_3_ therapy can mitigate the impact of I/R injury by diminishing cellular oxidative stress, enhancing the body’s natural antioxidant defense mechanisms, and lowering inflammation [11].

Ozone therapy involves the administration of ozone to the body using numerous methods, forms, and concentrations, such as ozone oxide [25,32]. These uses encompass techniques such as administering O_3_ gas directly through intravenous, intra-articular, or intramuscular injection, applying O_3_ oils or water to skin or mucosa surfaces, consuming ozonated water, and utilizing ozone in conjunction with carbon dioxide gasses [27,39,44,45,46,47].

While ozone and trimetazidine have been extensively researched as protective agents against organ damage resulting from ischemia–reperfusion, there is a limited amount of research on I/R-induced intestinal damage. Furthermore, none of these studies has been conducted comparatively or specifically focused on endoplasmic reticulum stress. The main purpose of this study is to find out how O_3_ therapy and trimetazidine might help reduce damage to the intestines caused by I/R by changing the endoplasmic reticulum stress response. Comparing the effects and possible protective mechanisms of these two therapy techniques is what the study aims to do. The goal is to help the development of new therapeutic approaches for treating intestinal I/R injuries.

## 2. Materials and Methods

Recep Tayyip Erdogan University Animal Experiments Local Ethical Committee approved the study (Rize, Turkey) (approval date: 26 April 2022, approval number: 2022/13).

### 2.1. Animals and Experimental Design

**Figure 1:**
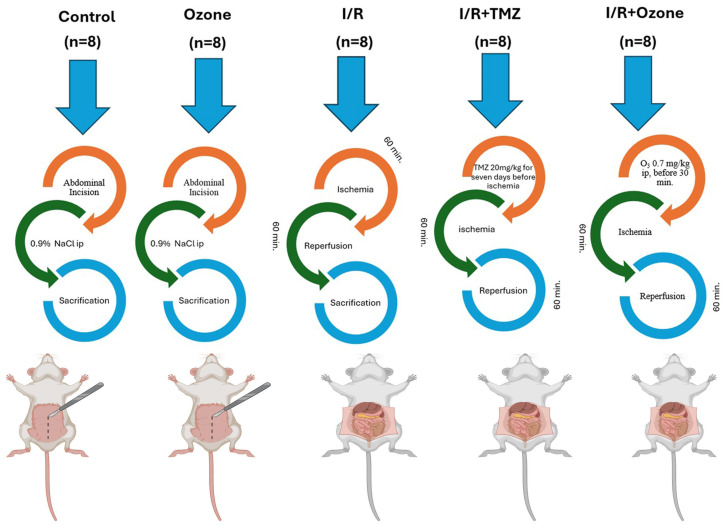
Experimental Study Design.

A total of 40 male adult Sprague Dawley rats, with an average weight of 250 ± 50 g, were utilized for the investigation. The rats were provided with tap water and conventional pellet meals on a 12 h day/night cycle at a temperature of 22 °C. The rats were randomly allocated into five groups, ensuring that each group had an equal number of rats with similar weights. Figure 1 shows the experimental study design.

The control group (C, n = 8) underwent laparotomy and exploration of the superior mesenteric artery (SMA), but no intestinal I/R injury was induced, and no drugs were administered. In addition, the rats were intraperitoneally injected with 1 mL of physiological serum.

The ozone group (O_3_, n = 8) received i.p. administration of a mixture of 0.7 mg/kg, 95% oxygen, and 5% ozone gas (O_3_-O_2_). This was performed in addition to laparotomy and SMA exploration. However, the I/R injury procedure was not applied.

The I/R group (I/R, n = 8) underwent a laparotomy procedure where the SMA was clamped using a silk suture and closed for 1 h. After that, the SMA was opened, and the intestines were allowed to reperfuse for 1 h.

The I/R+ trimetazidine group (I/R+ TMZ, n = 8) underwent laparotomy and intestinal I/R injury. The SMA was clamped with a silk suture and closed for 1 h, after which the SMA was opened, and the intestines were allowed to reperfuse for 1 h. Starting 1 week before the I/R injury, a dose of 20 mg/kg/day of trimetazidine was administered via oral gavage for 7 days. The final administration of TMZ occurred 30 min prior to the induction of I/R injury.

In the I/R + O_3_ group (I/R + O_3_, n = 8), the procedure involved performing a laparotomy and inducing intestinal I/R injury. This was performed by clamping the SMA with a silk suture for 1 h, followed by releasing the clamp and allowing the intestines to reperfuse for 1 h. An i.p. infusion of an O_3_-O_2_ gas combination (0.7 mg/kg) was administered 30 min prior to reperfusion.

Before the surgical procedure, all rats had a period of fasting overnight. Prior to all surgical procedures, anesthesia was induced in all rats by administering ketamine (50 mg/kg) (Ketalar, Eczacıbaşı, İstanbul, Turkey) and xylazine (10 mg/kg) (Kepro xylazine, Biopharm, Turkey) [11,14]. The abdominal wall was depilated, and the skin was sterilized using a 10% povidone–iodine solution (Isosol, Merkez Lab, Istanbul, Turkey). The occlusion model of the superior mesenteric artery (SMA) was implemented by making slight modifications to the approach proposed by Onal et al. [14]. The presence of pallor and a purplish hue in the intestines was recognized as a sign of ischemia, whereas the restoration of the intestinal wall to its original pink color was considered as a criterion for reperfusion. The ARRIVE guidelines were rigorously followed at every stage of the experiment [48,49]. To obtain the necessary intestine samples, all rats were euthanized using the intracardiac puncture procedure at the 1st hour of reperfusion. In order to examine the damage to the intestinal mucosa, histological, immunohistochemical, and biochemical analyses were conducted.

### 2.2. Chemicals and Drugs

Anesthesia was administered using ketamine hydrochloride (Ketalar, Eczacıbaşı, Istanbul, Türkiye) and xylazine hydrochloride (Kepro xylazine, Biopharm, Istanbul, Turkey). Trimetazidine 20 mg/kg/day (Vastarel 20 mg, SERVIER ILAÇ VE ARAŞTIRMA A.S., Istanbul, Türkiye) was used. The dosage of trimetazidine was established based on prior research [50,51]. All chemicals used in laboratory experiments were provided by Sigma Chemical Co. and Merck (Merck KGAa, Darmstadt, Germany).

### 2.3. Ozone Therapy Procedure

Ozone was produced using an ozone generator machine (Evozone Basic Plus, Reutlinger, Germany). The ozone generator machine regulates the rate of gas flow and the concentration of ozone in real time using an integrated UV spectrometer. The flow rate of ozone was maintained at a constant value of 3 L/min. The gas mixture consisted of 97% O_2_ and 3% O_3_, resulting in a concentration of 60 μg/mL. Each animal received an intraperitoneal administration of roughly 3.2–4.2 mL of the gaseous mixture. Tygon polymer tubes and single-use silicon-treated polypropylene syringes (which are resistant to ozone) were utilized during the reaction to guarantee the containment of O_3_ and maintain consistent concentrations. A single dosage of ozone was first delivered 30 min after ischemia. The duration and amount of ozone treatment were calculated based on previous investigations [11,13].

### 2.4. Biochemical Analysis

#### 2.4.1. Preparation of Tissue Homogenate

A solution containing 20 millimolar (mM) of sodium phosphate and 140 mM of potassium chloride was prepared in a volume of 1 L. The solution had a pH of 7.4. Subsequently, a volume of 1 mL of homogenization solution was introduced into 100 mg of tissue. The intestine tissue was then homogenized using a QIAGEN Tissue Lyser II (Qiagen corporation, 19300 Germantown Road Germantown, MD 20874, USA) homogenizer and subjected to centrifugation at 800× *g* for 10 min at a temperature of 4 degrees Celsius. The resulting supernatant was used to perform assays for total thiols (TTs) and thiobarbituric acid reactive substances (TBARSs).

#### 2.4.2. Determinations of Tissue Malondialdehyde (MDA) Levels (TBARS Assay)

The TBARS assay was conducted using the methodology outlined in the study by Ohkawa et al. [52]. A solution was produced by combining 200 µL of intestine tissue supernatant, 50 µL of 8.1% sodium dodecyl sulfate (SDS), 375 µL of 20% acetic acid (*v*/*v*) with a pH of 3.5, and 375 µL of 0.8% thiobarbituric acid (TBA). The mixture was vigorously agitated using a vortex mixer, and the reaction was allowed to undergo incubation in a water bath maintained at boiling temperature for a duration of 1 h. Following incubation, the sample was subjected to a cooling process in ice water for a duration of 5 min. Subsequently, it was centrifuged at a force of 750× *g* for a period of 10 min. The resultant pink hue was quantified using a spectrophotometer at a wavelength of 532 nm. The results were quantified in nanomoles per milligram of protein.

#### 2.4.3. Determination of Tissue Total Thiols (TTs), Glutathione (GSH) Levels

The TT group was determined using Ellman’s reagent [53]. First, 250 µL of intestine supernatant was combined with 1000 µL of 3 M Na_2_HPO_4_ and 250 µL of 5,5′-dithiobis (2-nitrobenzoic acid) (DTNB) (4 mg DTNB produced in 10 mL of 1% sodium citrate solution). The mixture was then vortexed, and the absorbance at 412 nm was measured. The results were determined using a pre-established standard curve of reduced glutathione ranging from 1000 µM to 62.5 µM, and the values were reported in nmol/mg protein.

### 2.5. Histopathological Analysis

Male Sprague Dawley rats were used to obtain small intestine tissue samples. The samples were trimmed to a volume of 1.5 cm^3^ and then placed in tissue tracking cassettes with lids. The small intestinal tissue sections were preserved by immersing them in a 10% phosphate-buffered formalin solution (Sigma Aldrich, Germany) for a period of 36 h. After the fixation process, small intestine tissue samples were collected using a tissue tracking device. The collection was performed following the routine histological tissue protocol, which involved dehydration using a series of increasing ethanol concentrations (50%, 70%, 80%, 90%, 96%, 100%). Mordanting was then carried out using two series of xylol. Following the clearing process, the small intestinal tissues were immersed in solid paraffin (Merck KGAa, Darmstadt, Germany) using a tissue embedding device (Leica 1150EGÜ, Leica Biosystem, Germany) through the application of paraffin embedding (Merck KGAa, Darmstadt, Germany). Sections of 4–5 µm in thickness were extracted from the blocks of small intestinal tissue using a rotary microtome (Leica RM2525, Lecia Biosystems, Germany). These sections were then stained with Harris hematoxylin and eosin G (H&E; Merck, GmbH, Darmstadt, Germany).

### 2.6. Immunohistochemical (IHC) Analysis

We employed C/EBP homologous protein (CHOP) (rabbit polyclonal, ab11419, Abcam, UK) and glucose-regulated protein-78 (GRP-78) primary antibody (rabbit polyclonal, ab1685, Abcam, UK) kits in our investigation to assess endoplasmic reticulum stress. Secondary antibody kits were utilized in conjunction with primary kits.

Sections of 2–3 µm in thickness were obtained from paraffin blocks of small intestine tissue using a rotary microtome (Leica RM2525, Lecia Biosystems, Germany). The small intestinal tissue sections were incubated with primary and secondary antibodies for 60 min following the deparaffinization and antigen retrieval process using the ISH/ICH apparatus (Bond Max, Leica Biosystems, Australia) as per the manufacturer’s guidelines. Following the application of antibodies, tiny slices of intestinal tissue were subjected to staining using diaminobenzidine tetrahydrochloride (DAB ultraview, Leica Biosystems, Germany) and Harris hematoxylin (Merck KGAa, Darmstadt, Germany).

### 2.7. Semi-Quantitative Analysis

The scoring of histopathological damage in intestine tissue was determined using the Intestinal Histopathological Damage Score (IHDS) under the categories of necrosis in germinal epithelial cells, fusion in villi, infiltrative areas, and hemorrhagic areas. This scoring system was based on studies of mesenteric artery ischemia and reperfusion (Table 1) [54,55]. A histopathologist, who was blinded to the study groups, graded 20 areas derived from 4 randomly chosen slides of small intestine tissue from each rat.

The histopathologist, who was blinded to the study groups, evaluated the enterocytes that exhibited CHOP/DDIT3 and GRP-78 positivity. The results of this analysis may be found in Table 2. The IHC positivity score was determined by randomly picking 20 distinct regions from 4 slides of each rat.

### 2.8. Statistical Analysis

The data acquired from biochemical, histological, and immunohistochemical analyses were assessed for their eligibility for a normal distribution using the Shapiro–Wilk test, skewness–kurtosis values, Q-Q plot, and Levene tests. The parametric data were computed by adding the mean to the standard deviation. The presence of a statistical difference between the groups was assessed using one-way ANOVA and Tukey HSD tests. The non-parametric data were computed using the median (25–75% interquartile range) and examined using the Kruskal–Wallis and Tamhane T2 tests. Significance was attributed to *p*-values that were less than 0.05.

## 3. Results

### 3.1. Biochemical Results

#### 3.1.1. Results of Tissue Malondialdehyde (MDA) Levels (TBARS Assay)

The MDA values obtained from the ileum tissues for the measurement of lipid peroxidation are presented in Table 3. Based on our findings, we did not observe any discernible distinction between the control group and the group exposed to ozone alone. In contrast, we found that the levels of MDA in the I/R group were considerably higher than those in the control group and the O_3_-only treatment group (*p* < 0.05). However, we observed a substantial decrease in MDA levels in the I/R + TMZ and I/R + O_3_ treatment groups compared to the I/R application group (*p* < 0.05). Nevertheless, the levels of MDA in both the I/R + TMZ and I/R + O_3_ therapy groups exhibited similarity, with no significant statistical difference seen.

#### 3.1.2. Results of Tissue Total Thiol (TT) Levels

Table 3 shows the measured glutathione (GSH) levels in rat ileum tissue. When assessing the antioxidant activity in ileum tissue, we found no significant disparity in GSH levels between the control group and the group treated with O_3_ alone. In contrast, we observed a substantial decrease in the GSH level in the group that had I/R application, as compared with both the control group and the group that received only O_3_ application (*p* < 0.05). However, we observed a substantial increase in the tissue level of GSH in the I/R + TMZ and I/R + O_3_ treatment groups compared with the I/R application group (*p* < 0.05). Furthermore, we observed significantly elevated GSH levels in the I/R + O_3_ treatment group as compared with the I/R + TMZ treatment group (*p* < 0.05).

### 3.2. Histopathological Results

**Figure 2:**
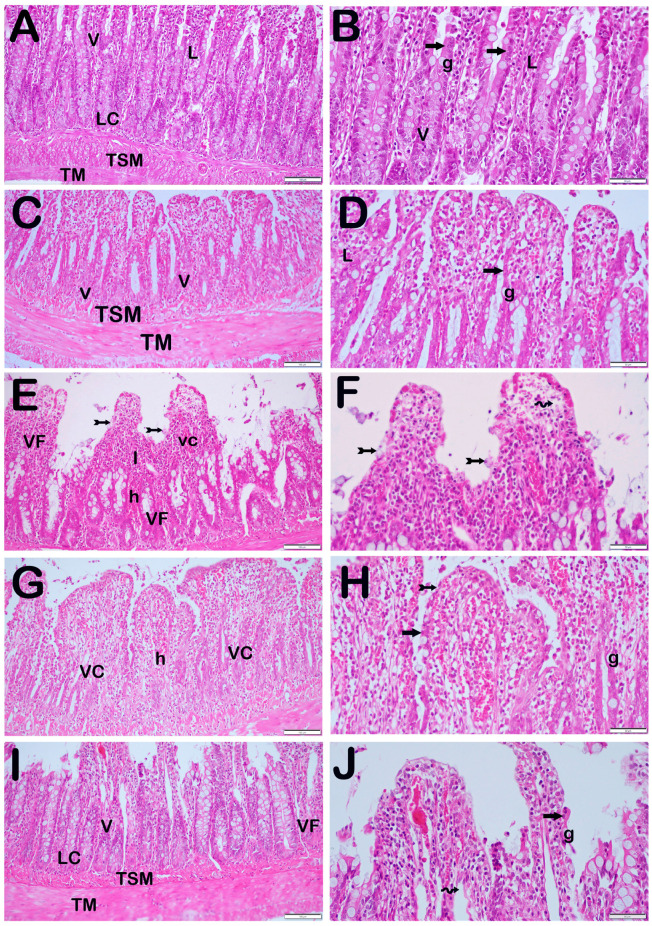
Representative Light Microscopic Screen Image of H&E-Stained Intestinal Tissue Sections. Tunica Submucosa (TSM), Tunica Muscularis (TM), Lamina Propria (L), Lieberkühn Crypts (LC), Goblet Cells (G), Enterocytes (arrow). (**A**(×20),**B**(×40)) Control Group**:** Normally structured epithelium consisting of enterocytes (arrow) and goblet cells (g) is observed. In addition, normally structured tunica submucosa (tsm) and tunica muscularis (tm) layers are observed (IHDS 1 (0–1)). (**C**(×20),**D**(×40)) O_3_ Group: The tunica mucosa layer consisting of enterocytes and goblet cells with a typical structure is observed (IHDS 1 (1–2)). (**E**(×20),**F**(×40)) I/R Group: Diffuse necrosis (tailed arrow) and villus fusions (vf) are observed in intestinal epithelial cells. In addition, Gruenhagens space extension (spiral arrow), vascular congestion (vc), hemorrhage (h), and widespread inflammations (I) in the lamina propria are observed (IHDS 9 (9–11)). (**G**(×20),**H**(×40)) I/R + TMZ Group: A decrease in necrotic enterocytes and villus fusions is observed. In addition, a reduction in hemorrhage, vascular congestion, and inflammation is observed (IHDS 4 (4–5.5)). (**I**(×20),**J**(×40)) I/R + O_3_ Group: In addition to the decrease in the number of necrotic enterocytes in intestinal epithelial cells, enterocytes with a widespread typical structure are observed. It is observed that there is a decrease in villus fusions, vascular congestion, and hemorrhage areas. In addition, it is observed that the infiltrative areas in the lamina propria have decreased (IHDS 1 (0–1)).

When the ileum tissue sections of the control and O_3_-only groups, stained with Harris hematoxylin and eosin G, were examined under a light microscope, we observed that there were villus structures with normal epithelial cells (Figure 2A,B, Table 4, IHDS: 1 (0–1), and Figure 2C,D, Table 4, IHDS: 1 (1–2), respectively). In the ileum sections of the I/R application group, we saw villus fusions along with a lot of necrotic epithelial cells. In addition, we found widespread infiltrative and hemorrhagic areas. Additionally, there were enlargements and capillary congestions in the lamina propria Gruenhagen space (Figure 2E,F, Table 4, IHDS: 9 (9–11)). On the other hand, in cross-sections of ileum tissue belonging to the I/R + TMZ treatment group, we found that there was a decrease in necrotic epithelial cells and villus fusions compared with the I/R application group (Figure 2G,H, Table 4, IHDS: 4 (4–5.5)). We also observed that infiltrative and hemorrhagic areas in the lamina propria decreased (Figure 2G,H, Table 4, IHDS: 1 (0–1)). Similarly, in the ileum sections of the I/R + O_3_ treatment group, villus fusions, necrotic epithelial cells, and infiltrative and hemorrhagic areas were significantly reduced compared with the I/R group (Figure 2I,J, Table 4, IHDS: 1 (0–1)). In addition, we found that histopathological damage, especially villus fusions (villus fusion score: 2 (2–2), 1 (0–1), respectively), was reduced in the I/R + O_3_ group compared with the I/R + TMZ group (Figure 2I,J, Table 4, *p* = 0.001, IHDS: 1 (0–1)).

### 3.3. Immunohistochemical (IHC) Results

#### 3.3.1. CHOP Positivity

**Figure 3:**
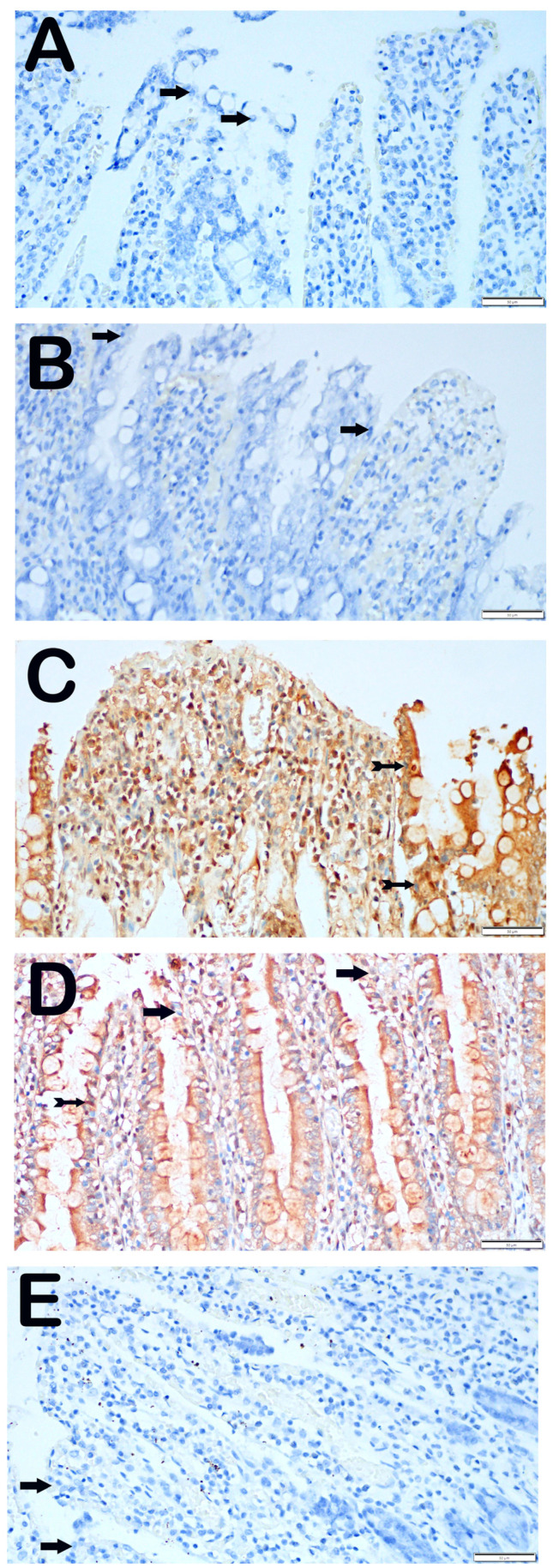
Representative Light Microscopic Screen Images of Intestinal Tissue Sections Incubated with CHOP Primary Antibody. (**A**(×40)) Control Group: It is observed that enterocytes with normal structure are immune-negative in terms of CHOP primary antibody (arrow, CHOP positivity score: 0 (0–0)). (**B**(×40)) O_3_ Group: Enterocytes with typical structure are observed to be immune-negative (arrow, CHOP positivity score: 0 (0–0)). (**C**(×40)) I/R Group: It is observed that the number of cells showing intense CHOP primary antibody positivity has increased (tailed arrow, CHOP positivity score: 2 (2–3)). (**D**(×40)) I/R + TMZ Group: It is observed that the number of cells showing positivity for intense CHOP primary antibody has decreased (tailed arrow, CHOP positivity score: 0.5 (0–1)). (**E**(×40)) I/R + O_3_ Group: Although the number of enterocytes showing positivity for the CHOP primary antibody has decreased, enterocytes with typical structures are commonly observed (arrow, CHOP positivity score: 0 (0–1)).

In the control group, we observed that the epithelial cells in the ileum tissue sections did not exhibit any indications of CHOP positivity when exposed to the CHOP primary antibody (Figure 3A, Table 5, CHOP positivity score: 0 (0–0)). Similarly, we found that the epithelial cells in the ileum sections of the ozone-treated group did not exhibit any immunopositivity, as indicated by their typical shape (Figure 3B, Table 5, CHOP positive score: 0 (0–0)). On the other hand, we observed a greater number of villus epithelial cells with a high intensity of CHOP positivity in the sections from the group that underwent I/R application compared with the control group (Figure 3B, Table 5, *p* = 0.001, CHOP positive score: 2 (2–3)). In contrast, we saw a decrease in the number of epithelial cells showing CHOP positivity in the ileum tissues of the I/R + TMZ treatment group compared with the I/R application group (Figure 3C, Table 5, *p* = 0.001, CHOP positivity score: 0.5 (0–1)). Similarly, we observed a significant reduction in the number of cells positive for CHOP in the ileum tissue sections of the I/R + O_3_ treatment group compared with the I/R application group (Figure 3D, Table 5, *p* = 0.001, CHOP positivity score: 0 (0–1)). Upon comparing the I/R + TMZ and I/R + O_3_ treatment groups, we observed that the sections of the I/R + TMZ group had a slightly higher degree of CHOP positivity. Nevertheless, this disparity did not have a statistically significant impact.

#### 3.3.2. GRP-78 Positivity

**Figure 4:**
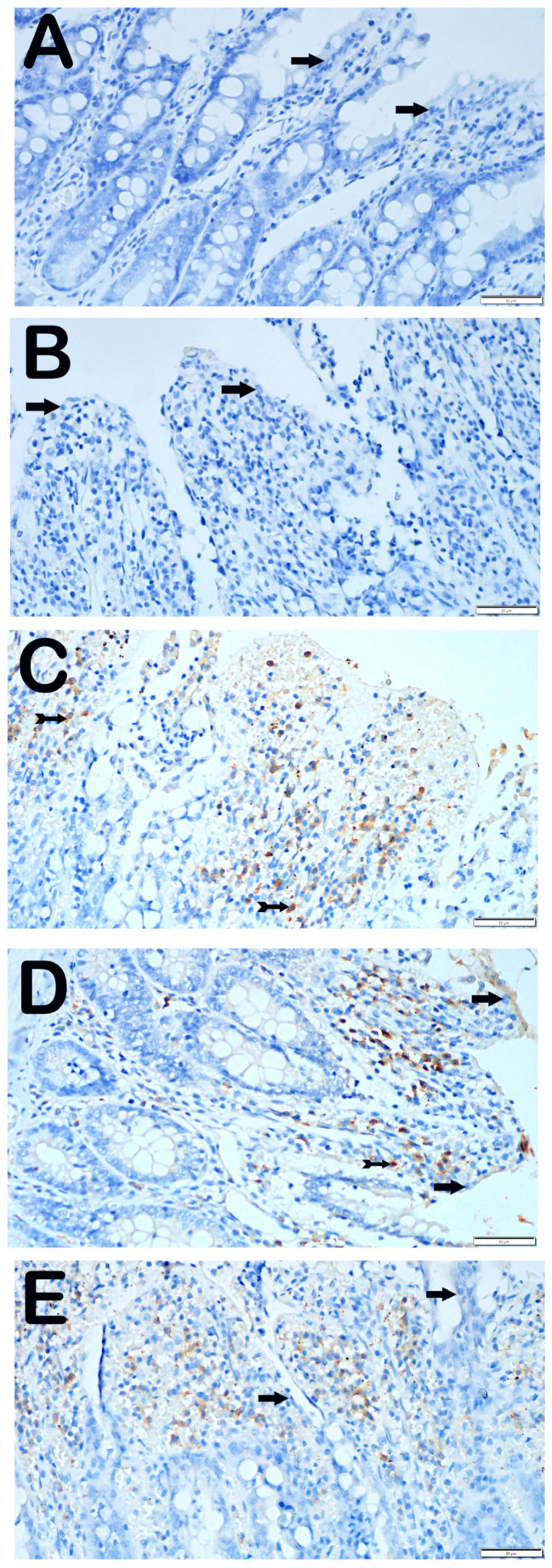
Representative Light Microscopic Screen Images of Intestinal Tissue Sections Incubated with GRP-78 Primary Antibody. (**A**(×40)) Control Group: Enterocytes with normal structure are observed to be immune-negative (arrow, GRP-78 positivity score: 0 (0–0.5)). (**B**(×40)) O_3_ Group: Enterocytes with typical structure are observed to be immuno-negative for GRP-78 primary antibody (arrow, GRP-78 positivity score: 0 (0–1)). (**C**(×40)) I/R Group: It is observed that the number of cells showing positivity for intense GRP-78 primary antibody has increased (tailed arrow, GRP-78 positivity score: 2 (1–2)). (**D**(×40)) I/R + TMZ Group: It is observed that the number of cells showing positivity for intense GRP-78 primary antibody has decreased (tailed arrow, GRP-78 positivity score: 1 (0–1)). (**E**(×40)) I/R + O_3_ Group: It is observed that the number of enterocytes showing positivity for GRP-78 primary antibody has decreased, but enterocytes with typical structure are commonly observed (arrow, GRP-78 positivity score: 0 (0–1)).

When examining the ileum tissue sections of the control group that were incubated with the GRP-78 primary antibody, it was discovered that the epithelial cells showed no immunological positivity (Figure 4A, Table 5, GRP-78 positive score: 0 (0–0.5)). Similarly, we observed that epithelial cells displaying the characteristic structure were not stained with GRP-78 in the ileum sections of the group that received only O_3_ application (Figure 4B, Table 5, GRP-78 positivity score: 0 (0–1)). In contrast, we found that the number of villus epithelial cells displaying strong GRP-78 positivity was higher in the sections of the I/R application group compared with the control group (Figure 4C, Table 5, *p* = 0.001, GRP-78 positivity score: 2 (1–2)). In contrast, we observed a reduction in the number of epithelial cells positive for GRP-78 in the ileum tissues of the I/R + TMZ treatment group compared with the I/R application group (Figure 4D, Table 5, *p* = 0.001, GRP-78 positive score: 1 (0–1)). In a similar manner, we noticed a substantial decrease in the number of cells positive for GRP-78 in the ileum tissue sections of the group treated with I/R + O_3_, compared with the I/R application group (Figure 4E, Table 5, *p* = 0.001, GRP-78 positivity score: 0 (0–1)).

## 4. Discussion

Intestinal ischemia–reperfusion damage is an intricate physiological process that can result in severe clinical outcomes [1]. This work investigated the molecular processes of I/R injury in a rat model induced by clamping the superior mesenteric artery. Additionally, we assessed the impact of ozone and trimetazidine therapies on this physiological process. The utilization of CHOP and GRP-78 immunohistochemical staining, as well as MDA and GSH biochemical investigations, in conjunction with histopathological analyses, facilitated the assessment of the magnitude of I/R damage in intestinal tissues and the involvement of ER stress. The results of our investigation demonstrated that both treatments mitigated I/R injury, with O_3_ therapy exhibiting slightly superior efficacy compared to TMZ therapy. The antioxidant and anti-inflammatory qualities of O_3_ therapy make it effective in lowering ER stress, as demonstrated by these studies.

Ischemia–reperfusion injury causes significant structural and chemical alterations, particularly in the intestinal tissue [11,13]. The histopathological and biochemical indicators, including MDA and GSH levels, utilized in this study are essential for evaluating the severity of the damage and the effectiveness of the treatments. MDA, a biomolecule known as malondialdehyde, serves as a reliable sign of lipid peroxidation. It is a crucial marker for assessing cellular harm and oxidative stress [56]. The observed elevation of MDA levels and reduction of GSH levels in the I/R group imply an augmentation of oxidative stress in the intestinal tissue and a decline in antioxidant defense. Previous research has revealed similar outcomes [14]. Overproducing ROS in tissues leads to I/R injury. ROS damage cells by encouraging lipid peroxidation [57]. This mechanism results in the reduction of antioxidant molecules like GSH and an increase in oxidative stress [57]. These data validate that intestinal I/R leads to oxidative harm and diminished cellular antioxidant capability. However, the observed decrease in MDA levels in the I/R + TMZ and I/R + O_3_ groups indicates that these therapies effectively mitigate oxidative damage. The results indicate that O_3_ has the ability to decrease lipid peroxidation, especially in the context of O_3_ therapy [14].

Glutathione, functioning as an intracellular antioxidant, plays a crucial role in managing oxidative stress [57]. Our study revealed a decline in GSH levels in the I/R group, suggesting that the body’s ability to defend against oxidative stress was depleted and its protection against oxidative damage was diminished. The rise in GSH levels in the I/R + TMZ and I/R + O_3_ groups indicates that the therapies enhance the body’s ability to counteract oxidative damage and reduce oxidative stress. Specifically, the observation that the I/R + O_3_ group exhibited increased GSH levels compared with the I/R + TMZ group suggests that O_3_ could be a more potent modulator of the glutathione pathway.

Existing literature has established that O_3_ exhibits protective properties in different inflammatory and oxidative circumstances owing to its anti-inflammatory and antioxidant activities [25,30]. Ozone’s ability to enhance GSH levels can be attributed to its ability to regulate the redox equilibrium in biological systems and boost cellular antioxidant defense [33,44]. However, it was shown that the biochemical alterations induced by I/R injury were rectified in the groups receiving therapy with TMZ. The reduction in MDA levels and the elevation in GSH levels demonstrate the impact of TMZ on diminishing cellular oxidative stress. The findings of prior studies have also shown comparable results [58]. TMZ is recognized for its ability to enhance energy metabolism and decrease the formation of ROS via boosting mitochondrial processes [51].

There was no notable disparity in the levels of MDA and GSH between the groups that received O_3_ treatment and the control group. This indicates that O_3_ by itself does not possess antioxidant properties, but it may be able to reverse metabolic alterations following harm caused by I/R. Specifically, it was noted that the levels of GSH were elevated in the I/R + O_3_ group in comparison to the I/R + TMZ group. This indicates that O_3_ enhances the cellular antioxidant defenses by augmenting the antioxidant capacity. The findings of this study provide evidence for the possible application of O_3_ as an antioxidative therapy. Additionally, the results demonstrate that TMZ also possesses protective properties against oxidative stress.

This study assessed the occurrence of intestinal I/R injury in rats by examining histological characteristics, including necrosis, villus fusion, infiltrative regions, and hemorrhagic areas in intestinal epithelial cells. The control group and the O_3_-only group displayed normal histopathological structure, whereas the I/R group showed significant damage. Specifically, the I/R group had a large increase in necrotic epithelial cells, villus fusions, infiltrative areas, and hemorrhagic areas. The findings suggest that I/R injury leads to significant alterations in the structure of the intestinal tissue, with particularly extensive damage occurring in the villi and lamina propria.

The study demonstrated that the administration of TMZ and O_3_ therapies resulted in significant improvements in I/R injuries. Administration of TMZ and O_3_ therapy following I/R resulted in a substantial decrease in histological injury and reinstated the original architecture of the intestinal tissue. Specifically, it was noted that the O_3_ therapy groups had fewer instances of villus fusions and hemorrhagic areas compared to the TMZ treatment groups. These data indicate that O_3_ may be more efficient in preventing I/R injury and promoting the repair of intestinal tissue compared to TMZ. This difference could perhaps arise from ozone’s capacity to regulate inflammatory mechanisms and directly mitigate oxidative damage. Furthermore, O_3_ is believed to enhance cellular adaptability and repair mechanisms by directly influencing intracellular signaling pathways [28,33].

The findings align with other research that emphasizes the positive impact of O_3_ on endothelial function and microcirculation [13]. For instance, a study conducted by Onal et al. discovered that O_3_ therapy exhibited a defensive impact on intestinal I/R injury and facilitated the process of tissue recovery [14]. In a similar vein, a further study conducted by Tetik et al. showed that TMZ treatment effectively decreased I/R injury and partially maintained the structural integrity of intestinal tissue [59]. These findings suggest that O_3_ may protect the intestines by lowering oxidative stress and enhancing microvascular circulation [13]. On the other hand, while TMZ treatment similarly decreased damage, it was not as efficacious as O_3_. TMZ enhances cellular energy metabolism, increasing resistance to ischemia [51]. However, the O_3_ treatment in this study resulted in more noticeable improvements in cell morphology.

**Figure 5:**
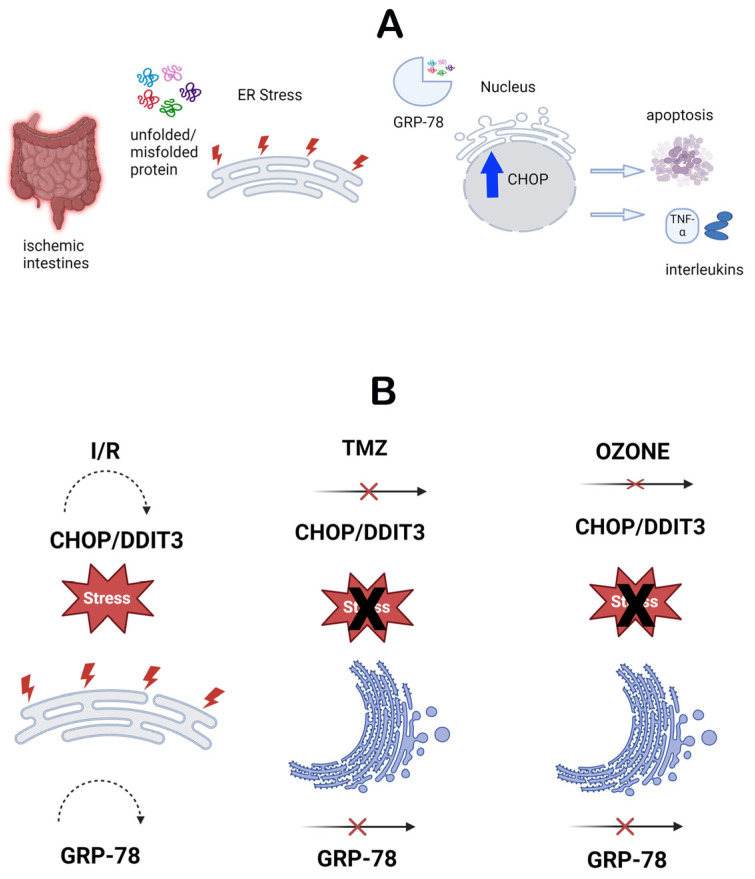
(**A**). Schematic representation of the endoplasmic reticulum stress mechanism. (**B**). Schematic representation of the effects of TMZ and O_3_ on the endoplasmic reticulum stress mechanism.

Endoplasmic reticulum stress refers to the activation of the cellular stress response caused by the buildup of proteins in the ER due to folding problems (Figure 5A). This stress is characterized by the presence of molecules such as CHOP and GRP-78 [60]. These molecules have a significant role in indicating the presence of ER stress and are linked to several clinical disorders [20]. The molecules were assessed by IHC analysis in this study. The control and O_3_-only groups exhibited similar levels of CHOP and GRP-78 immunopositivity. Nevertheless, the I/R group had elevated levels of immunopositivity for both molecules, indicating that damage caused by I/R in the intestinal tissue induces ER stress and promotes the excessive production of these molecules. This demonstrates that ER stress is successfully triggered in this particular model. This discovery suggests that ischemia and reperfusion lead to significant stress and harm to cells, leading to malfunction of the ER. However, there was a notable reduction in the presence of CHOP and GRP-78 in the I/R + TMZ and I/R + O_3_ groups. The results indicate that both O_3_ and TMZ have the ability to regulate pathogenic processes related to ER stress. Specifically, the application of O_3_ demonstrated a more significant reduction in CHOP and GRP-78 levels when compared to the I/R + TMZ group. This implies that O_3_ may exert a more potent influence on ER stress. It is possible that ozone can mitigate ER stress by enhancing the ability to fold proteins or decreasing the signals that lead to cell death caused by ER stress (Figure 5B).

These findings indicate that O_3_ therapy may be a viable option for treating disorders associated with ER stress. Moreover, these findings are based on data that promote a more comprehensive comprehension of ER stress pathways and the exploration of the impacts of manipulating these pathways on disease mechanisms. Exploring the mechanisms by which O_3_ and TMZ modulate ER stress in this situation may lead to the development of techniques that enhance the efficacy of these therapies. Simultaneously, our findings suggest that therapeutic interventions aimed at addressing ER stress could be a potentially effective method for treating intestinal I/R injury.

In this study, the effects of O_3_ therapy on I/R injury were evaluated by administering a single-dose intraperitoneal administration 30 min before reperfusion. Nevertheless, numerous studies in the literature have documented varying O_3_ application times, doses, and administration routes [14,28,31,32,34,39,61]. It is crucial to compare these various application methods in order to identify the most effective treatment options. The existing research on O_3_ therapy encompasses a range of techniques on the timing, dose, and manner of application [32,39]. These approaches can exert a substantial influence on the effectiveness of the treatment.

Our ozone application approach has both similarities and variations when compared to numerous studies in the literature. Timing the application of O_3_ before reperfusion is a crucial element. Due to its unpredictable nature, I/R injury is not easily anticipated in a clinical setting. Our investigation found that applying O_3_ 30 min prior to the occurrence of I/R damage is the optimal method. This approach allows for early intervention when the damage is discovered and gives enough time for cellular adaptive mechanisms to be triggered [11,13]. A study conducted by Isık et al. revealed that the administration of ozone 45 min before reperfusion successfully stimulated antioxidant defense mechanisms [13]. Nevertheless, we also have apprehensions that applications applied far in advance may diminish their efficacy. Hence, it is crucial to examine the individual experimental settings and desired therapeutic outcomes when establishing the ideal timing for O_3_ treatment.

Furthermore, the amount of O_3_ used is also a crucial factor in determining the effectiveness of the treatment. The selection of a single and low-dose application in this investigation aligns with prior studies in the literature and has been generally determined to be both safe and efficacious [11,13]. Research in the literature indicates that small amounts of O_3_ have antioxidant properties and can decrease cellular harm [25,30,39]. However, there have been reports indicating that prolonged exposure to O_3_ and high levels of exposure can cause oxidative effects and could lead to tissue damage [25]. Furthermore, given the clinical nature of I/R injury, there is often insufficient time to administer repeated doses. It is important to note that altering the dosage, whether increasing or reducing it, might result in varying biological effects, particularly when the medication is taken over a lengthy period of time or on a regular basis. Hence, additional dose–response studies are required to ascertain the most effective dosage of O_3_ therapy.

Ultimately, the manner in which O_3_ is administered is a crucial determinant of the treatment’s efficacy. The intraperitoneal route of administration was favored in our investigation. Furthermore, apart from the intraperitoneal route, other investigations have documented the administration of O_3_ using intravenous, subcutaneous, intra-articular, or rectal methods [10,28,30,35,36,38,42,45,47,62]. Intraperitoneal administration is a technique that enhances the bioavailability of O_3_ and allows it to reach the desired tissue [11]. Additionally, it enables the quick attainment of both local and systemic effects, making it a preferable approach in instances of acute I/R [11]. Prior research has demonstrated that the administration of O_3_ into the peritoneal cavity leads to a swift decrease in oxidative stress and inflammation within cells by quickly entering the bloodstream [11,61]. A single-dosage administration of O_3_ utilized in our investigation may initiate this prompt impact, however, recurrent administrations may also provide sustained safeguarding. Alternatively, the use of O_3_ either locally or systemically has been found to be helpful in various stress circumstances [36]. The various methods of administering O_3_ can result in alterations in the bioavailability and processes by which O_3_ acts. Every method of administering a substance has its own set of benefits and drawbacks, and these variables must be taken into account when deciding on the most effective method for clinical use.

Consequently, the use of O_3_ therapy through intraperitoneal administration, given as a single dosage prior to reperfusion, has the potential to be successful in the intestinal I/R model. Nevertheless, further comparative research is necessary to comprehend the potential impact of varying dosages and frequencies of administration on these outcomes. By implementing O_3_ therapy in clinical applications, we can enhance its effectiveness and expand its therapeutic range.

This study presents crucial findings regarding the efficiency of O_3_ and TMZ treatment for intestinal I/R injury. Several studies in the literature have examined the impact of ozone or trimetazidine treatment on harm caused by ischemia–reperfusion in various organs. Currently, there is limited research in the literature exploring the impact of ozone and no research examining the effects of trimetazidine on intestinal damage caused by ischemia–reperfusion, which is the focus of our investigation. Furthermore, our study examined these two drugs through a direct comparison. An essential aspect of our work is that it investigated all these impacts by means of endoplasmic reticulum stress. However, it is important to note that the study also has some drawbacks. The rat model utilized in our work is not a precise representation of human intestinal tissue; hence, the direct relevance of the findings to people may be restricted. Human clinical trials are necessary to ascertain if the findings shown in rats can be reproduced in humans. Furthermore, it is necessary to further refine the therapy protocols and dosages employed in this study before directly applying them to clinical settings. Variations in elements such as the length of treatment, the amount of medication administered, and the methods used for application can influence the way outcomes are understood. Hence, additional clinical trials are required to ascertain the most effective treatment protocols and dosages. Furthermore, it should be noted that the markers employed in this work to evaluate ER stress may not comprehensively capture the intricate mechanisms associated with ER stress. Not considering additional indicators of ER stress and the related signaling cascades could result in an incomplete analysis of the study. Hence, it is crucial to investigate several ER stress indicators and signaling pathways in the next research. Ultimately, this study only assessed the effects of O_3_ and TMZ treatments. Nevertheless, it is crucial to assess the impacts of alternative therapeutic agents in treating intestinal I/R injury. For instance, it is important to evaluate the efficacy of alternative therapeutic approaches such as antioxidants, anti-inflammatory medications, or mitochondrial protectants. Given these limitations, it is crucial to interpret the findings cautiously and do additional studies to address the gaps in these areas.

## 5. Conclusions

This study aimed to assess the impact of ozone therapy and trimetazidine administrations on endoplasmic reticulum stress. An experimental model of intestinal I/R was created in rats for this purpose. Immunohistochemical stainings for CHOP and GRP-78, as well as biochemical analysis of MDA and GSH, were conducted to investigate the impact on cellular stress and antioxidant defense mechanisms. The results indicated that both O_3_ injection therapy and TMZ were successful in minimizing intestinal I/R injury and alleviating ER stress during the injury. The greater increase in GSH levels caused by ozone therapy compared with TMZ suggests that O_3_ may have a broader antioxidant effect. These findings provide evidence for the prospective advantages of utilizing O_3_ therapy in the treatment of intestinal I/R injury. They also indicate that O_3_ should be examined in bigger clinical trials in the future as a therapeutic agent that can disrupt ER stress. Nevertheless, it is crucial to validate these findings with more extensive and enduring investigations. The findings of this study could potentially aid in the advancement of novel therapeutic approaches for the pathophysiology of ischemia–reperfusion.

## Figures and Tables

**Table 1:** Intestinal Histopathological Damage Score Modified By Parks and Chiu (IHDS).

Score	Findings
Villus Epithelial Cell Necrosis	
0	≤5%
1	6–25%
2	26–50%
3	≤50%
Villus Fusion	
0	≤5%
1	6–25%
2	26–50%
3	≥50%
Lamina Propria Inflammation	
0	≤5%
1	6–25%
2	26–50%
3	≥50%
Gruenhagens Space Extension with Capillary Congestion	
0	≤5%
1	6–25%
2	26–50%
3	≥50%
Hemorrhage	
0	≤5%
1	6–25%
2	26–50%
3	≥50%

**Table 2:** Immunohistochemical Positivity Score.

Score	
	None (less than 5%)
1	Mild (from 6–25%)
2	Moderate (from 26–50%)
3	Severe (from 51–75%)
4	Very Severe (more than 76%)

**Table 3:** Biochemical Analysis Results (Mean ± Std. deviation).

Groups	MDA Levels (nmol/mg) (Mean ± Std. Deviation)	GSH Levels (nmol/mg) (Mean ± Std. Deviation)
Control	3.86 ± 0.52	34.97 ± 0.86
O_3_	3.86 ± 0.47	36.02 ± 0.59
I/R	6.78 ± 0.13 ^a,b^	26.54 ± 0.70 ^a,b^
I/R + TMZ	4.04 ± 0.47 ^c^	28.63 ± 0.93 ^d^
IR + O_3_	3.96 ± 0.93 ^c^	30.54 ± 1.22 ^c,e^

^a^ *p* = 0.001 versus control group, ^b^ *p* = 0.008 versus O_3_ group, ^c^ *p* = 0.001 versus I/R group, ^d^ *p* = 0.003 versus I/R group, ^e^ *p* = 0.008 versus I/R + TMZ group, Kruskal–Wallis/Tamhane T2 test.

**Table 4:** IHDS Results (Median (25%–75% interquartile range)).

Groups	Villus Epithelial Cell Necrosis	Villus Fusion	Lamina Propria Inflammation	Gruenhagens Space Extension with Capillary Congestion	Hemorrhage	IHDS
Control	0 (0–0)	0 (0–0)	0 (0–0)	0 (0–0)	0 (0–0)	1 (0–1)
O_3_	0 (0–0.5)	0 (0–0.5)	0 (0–0.5)	0 (0–0)	0 (0–0)	1 (1–2)
I/R	2 (2–3) ^a,b^	3 (2–3) ^a,b^	1 (1–2) ^a,b^	2 (1–2) ^a,b^	1 (0.5–1) ^a^	9 (9–11) ^a,b^
I/R + TMZ	1 (1–1) ^c^	2 (2–2) ^c^	0 (0–0) ^c^	0 (0–1) ^c^	1 (0–1) ^d^	4 (4–5.5) ^a,c^
I/R + O_3_	1 (0–1) ^c^	1 (1–1) ^c,f^	0 (0–0) ^c^	0 (0–0.5) ^c^	0 (0–0.5) ^c^	1 (0–1) ^a,e,f^

^a^ *p* = 0.001 versus control group, ^b^ *p* = 0.001 versus O_3_ group, ^c^ *p* = 0.001 versus I/R group, ^d^ *p* = 0.05 versus I/R group, ^e^ *p* = 0.015 versus I/R group, ^f^ *p* = 0.001 versus I/R + TMZ group, Kruskal–Wallis/Tamhane T2 test.

**Table 5:** Immunohistochemical Analysis Results (median (interquartile range 25%–75%)).

Groups	CHOP/DDIT3 Positivity Score	GRp-78 Positivity Score
Control	0 (0–0)	0 (0–0.5)
O_3_	0 (0–0)	0 (0–1)
I/R	2 (2–3) ^a,b^	2 (1–2) ^a,b^
I/R + TMZ	0.5 (0–1) ^c^	1 (0–1) ^c^
I/R + O_3_	0 (0–1) ^c^	0 (0–1) ^c^

^a^ *p* = 0.001 versus control group, ^b^ *p* = 0.001 versus O_3_ group, *^c^ p* = 0.001 versus I/R group, Kruskal–Wallis/Tamhne T2 test.

## Data Availability

All data generated for the manuscript are included in the study and are available upon request.
